# Metformin and phenethyl isothiocyanate combined treatment *in vitro* is cytotoxic to ovarian cancer cultures

**DOI:** 10.1186/1757-2215-5-19

**Published:** 2012-07-10

**Authors:** Daniel K Chan, W Keith. Miskimins

**Affiliations:** 1 Cancer Biology Research Center, Sanford Research/USD, 2301 East 60th Street-North, Sioux Falls, SD, 57104, USA

**Keywords:** Metformin, Phenethyl isothiocyanate, Ovarian cancer

## Abstract

**Background:**

High mortality rates in ovarian cancer are largely a result of resistance to currently used chemotherapies. Expanding therapies with a variety of drugs has the potential to reduce this high mortality rate. Metformin and phenethyl isothiocyanate (PEITC) are both potentially useful in ovarian cancer, and they are particularly attractive because of their safety.

**Methods:**

Cell proliferation of each drug and drug combination was evaluated by hemacytometry with Trypan blue exclusion or Sytox green staining for cell death. Levels of total and cleaved PARP were measured by Western blot. General cellular and mitochondrial reactive oxygen species were measured by flow cytometry and live cell confocal microscopy with the fluorescent dyes dihydroethidine and MitoSOX.

**Results:**

Individually, metformin and PEITC each show inhibition of cell growth in multiple ovarian cancer cell lines. Alone, PEITC was also able to induce apoptosis, whereas metformin was primarily growth inhibitory. Both total cellular and mitochondrial reactive oxygen species were increased when treated with either metformin or PEITC. The growth inhibitory effects of metformin were reversed by methyl succinate supplementation, suggesting complex I plays a role in metformin's anti-cancer mechanism. PEITC's anti-cancer effect was reversed by N-acetyl-cysteine supplementation, suggesting PEITC relies on reactive oxygen species generation to induce apoptosis. Metformin and PEITC together showed a synergistic effect on ovarian cancer cell lines, including the cisplatin resistant A2780cis.

**Conclusions:**

Here we show that when used in combination, these drugs are effective in both slowing cancer cell growth and killing ovarian cancer cells *in vitro*. Furthermore, the combination of these drugs remains effective in cisplatin resistant cell lines. Novel combinations such as metformin and PEITC show promise in expanding ovarian cancer therapies and overcoming the high incidence of cisplatin resistant cancers.

## Background

Ovarian cancer continues to have a disturbing discrepancy in incidence to mortality rate. While ovarian cancer trends as eighth in overall incidence among women in the United States, ovarian cancer mortality is significantly higher and ranks fifth in female cancer deaths. The difference in incidence to mortality rates is a combined result of poor ovarian cancer screening and rapid development of resistance to current chemotherapeutics. Poor ovarian cancer screening is inherent to its non-specific initial symptoms, and CA-125 and transvaginal ultrasound provide no additional benefit in reducing mortality [1]. Expanding available chemotherapies may improve the long term survival of ovarian cancer patients by overcoming the resistance to platinum based therapies and providing alternate mechanisms for cancer cell death. Metformin and PEITC have both been individually identified as potential ovarian cancer therapies [2-5]. This work examines the combination of metformin and PEITC as a novel treatment combination.

The exact mechanisms for the anti-cancer effects of both metformin and PEITC are not entirely clear. However, several groups have found both metformin and PEITC to affect complexes within the mitochondrial electron transport chain [6,7]. While cancer cells have an altered metabolism that heavily utilizes glycolysis, even in the presence of oxygen, mitochondrial oxidative metabolism is still necessary to generate intermediates needed for cell replication and reducing agents to deal with oxidative stress. This potential inhibition of complexes in the electron transport chain may increase reactive oxygen species (ROS) within the cell, especially in the mitochondria, leading to damage and eventually cancer cell death.

The mechanism of cisplatin resistance in ovarian cancer is not precisely known, but may be due to reduced uptake [8]. Some research suggests cisplatin uptake is dependent on the copper transporter CTR1 [9]. Metformin is highly concentrated in cells, likely through OCT1 [10], and has not been found to be affected by changes that occur in cisplatin resistant cell lines. PEITC is a lipophilic compound that does not rely on an active transporter and can directly cross the cell membrane. When taken orally in milligram amounts, peak PEITC plasma levels based on HPLC analysis were measured in the low micromolar range [11].

After being associated with lower cancer incidence in diabetic patients [12], metformin has been studied in a variety of cancers as a potential therapy. In breast cancer cells metformin has been found to induce cell death and improve the efficacy of existing therapies [13,14]. Previous research with metformin in ovarian cancer has suggested that mechanisms in addition to AMPK activation are responsible for inhibition of cell growth [3]. Metformin's partial inhibition of complex I, NADH dehydrogenase, has been identified as an effect potentially upstream of AMPK activation [7]. This mechanism is also interesting because it may provide an AMPK independent pathway for metformin to damage cancer cells through ROS production and mitochondrial damage.

PEITC gained interest as a potential anti-cancer agent based on findings of lower cancer incidence in people with diets high in cruciferous vegetables [15]. With PEITC specifically, preclinical animal models also associated the drug with decreased carcinogenesis [16,17]. Early studies of PEITC in transformed ovarian epithelial cells also found ROS generation to mediate cell death [5]. This ROS generation was later found to be a result of complex III, cytochrome bc1 complex, inhibition in colorectal cancer cells [6]. These drugs which work in alternative pathways from cisplatin may provide additional therapeutic options in ovarian cancer.

## Methods

### Reagents

Metformin, PEITC, cisplatin, and most other reagents were purchased from Sigma Aldrich (St. Louis, MO). Tissue culture media, DMEM and RPMI were purchased from Thermo-Fisher Scientific (Waltham, MA). Fetal bovine serum (FBS) was purchased from Atlanta Biologicals (Atlanta, GA). Fluorescent dyes: MitoSOX, 2’,7’-dichlorodihydrofluorescein (DCF), dihydroethidine (DHE), dihydrorhodamine 123 (DHR), Sytox green, and MitoTracker Green were all purchased from Invitrogen (Grand Island, NY). Antibodies to detect AMPK, PARP, cleaved PARP were purchased from Cell Signaling Technology (Beverly, MA). Antibodies to detect MnSOD were purchased from BD Pharmingen (San Diego, CA).

### Cell culture

Cell lines were incubated in a 37 C, 5% CO2 humidified chamber. OVCAR3, CAOV3, SKOV3, and PA-1 were obtained from ATCC. A2780 and A2780cis were received as a kind gift from Dr. Subhash Chauhan. OVCAR3, CAOV3, SKOV3, and PA-1 were maintained in DMEM supplemented with 10% fetal bovine serum, 100 units/mL penicillin, 100 μg/mL streptomycin, and 250 ng/mL amphotericin B. A2780 was maintained in RPMI with 10% FBS, 100 units/mL penicillin/streptomycin, and 250 ng/mL amphotericin B. A2780cis cells were maintained in complete RPMI media supplemented with 1 μM cisplatin every 3rd passage to maintain selection for cisplatin resistance.

### Cell counting

Multiple methods were used in cell counting. For cells counted by glass hemacytometer with Trypan blue exclusion, adherent cells were harvested by trypsinization. For live and dead cell counts, media was also collected and added to the cell suspension. Cells were pelleted by centrifugation (200 x g) and each sample was resuspended in an equal volume. Samples were then mixed with an equal volume of 0.4% Trypan blue and counted using a glass hemacytometer. Cells excluding Trypan blue were counted as live, stained cells were counted as dead. Sytox green (Invitrogen) fluorescent dye was also used for dead cell analysis. In a 96-well plate format, cells were plated in phenol red free media 24 hours prior to treatment with drugs of interest. At desired time points, Sytox green dye was added to wells at 1 μM final concentration and incubated for 10 minutes to stain DNA of dead cells. Fluorescence was measured using a Spectramax M5 fluorescence plate reader at excitation 485, emission 530, cutoff 515. Total cell fluorescence was measured by adding Triton X-100 at a final concentration of 0.1% to all wells, incubating for 10 minutes, and remeasuring Sytox fluorescence.

### Anchorage dependant colony formation assay

Cells were plated at 300–500 cells per 100 mm dish 24 hours before beginning treatment conditions. Cells were allowed to grow for 7–10 days before fixation. Colonies were fixed using 70% ethanol, and stained using 1% crystal violet dissolved in 10% ethanol. Number of colonies was obtained using image analysis software (AlphaInnotech). Colonies were defined as a minimum of 50 cells in a group.

### Western blotting

Samples were harvested in an SDS based buffer, sonicated and heated before running. Proteins were separated by SDS-PAGE and transferred to PVDF membrane by semi-dry transfer apparatus. Antibodies for detection of proteins of interest were diluted in blocking buffer, 5% BSA in TBST. Signal was detected using HRP-conjugated secondary antibodies and Amersham ECL prime detection reagent (GE healthcare). Exposures were captured either by photographic film or CCD camera imaging system (UVP).

### Flow cytometry for oxidative stress

After varying treatment periods, adherent cell cultures were detached with trypsin and transferred to culture tubes as a cell suspension. Cells were then incubated with fluorescent dyes to determine levels of oxidative stress products. MitoSOX (Invitrogen) was used to measure mitochondrial specific superoxide. Dihydroethidine (DHE) was used to measure total cellular superoxide. Absorbance was measured with the FL2 channel (Ex / Em) of an Accuri C6 flow cytometer. Data was collected and analyzed using Cflow Plus software (BD-Accuri).

### Live cell confocal microscopy

Cells were cultured on a chambered coverglass (Thermo scientific) in phenol red free DMEM with 10% FBS. After completing desired drug treatment courses, cells were stained with dyes of interest in phenol red free DMEM with 5% FBS. MitoSOX was used at 5 μM final concentration and MitoTracker green at 100 nM final concentration. Cells were incubated with dye for 15 minutes at 37 °C, and then washed twice with PBS. Phenol free DMEM with 5% FBS was used during live cell imaging. An Olympus FV1000 imaging system on an Olympus IX81 inverted microscope with heated 5% CO_2_ stage chamber was used for image capture. Image processing and analysis was completed using Olympus FluoView software.

### Statistics

Error bars displayed are standard deviations. Comparison of two groups was done using a two-tailed student’s t-test. Analysis of multiple groups was performed by ANOVA or Chi-squared test using R, the statistical computing program.

CalcuSyn (BioSoft) was used to calculate combination index values for drug combinations. Drug combinations were given at constant ratios and effect measured was either cell death or cell growth inhibition.

## Results

### Metformin is cytostatic, PEITC is cytotoxic in multiple ovarian cancer lines

**Figure 1:**
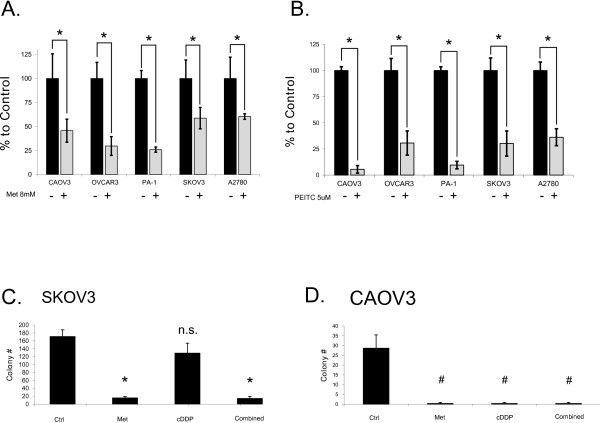
**Individual effects of metformin and PEITC on ovarian cancer lines*in vitro.*A**. Metformin growth inhibition of multiple human ovarian cancer cell lines after 48 hour treatment with control (H_2_O vehicle) 8 mM metformin. **B**. PEITC growth inhibition of multiple human ovarian cancer cell lines after 24 hour treatment with control (DMSO vehicle) or 5 μM PEITC. Both metformin and PEITC induce a significant decrease in cell proliferation across all tested cell lines. Cell proliferation and growth inhibition was measured using hemacytometry with Trypan blue exclusion. **C**. Anchorage dependant colony formation assay on cisplatin resistant SKOV3 line shows significantly fewer colonies in metformin [5 mM] compared to cisplatin [5uM] treatment. **D**. In CAOV3, metformin is just as effective as cisplatin in reducing colony formation. * p < 0.01; # p < 0.05; n.s. = not significant.

Across many human epithelial ovarian cancer cell lines, metformin was found to significantly lower cell proliferation (Figure 1A). This effect was greater over longer treatment periods (Compare 48 hour treatment Figure 1A and 24 hour treatment 5A below). Live cell proliferation was measured using a glass hemacytometer and Trypan blue exclusion. With drugs of interest that target cellular metabolism, direct live cell counting using a glass hemacytometer is preferred over assays such as the MTT which rely on cellular respiration to indirectly measure number of cells. In addition to inhibiting cell growth, anchorage dependant colony formation was strongly inhibited by metformin (Figure 1C and 1D). In the cisplatin resistant cell line SKOV3, metformin was still effective at preventing colony formation (Figure 1C).

**Figure 2:**
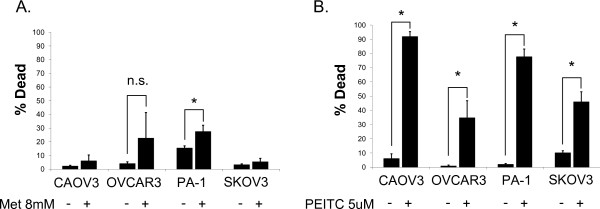
**Individual cell death effects of metformin and PEITC.A**. Metformin treatment [8 mM] on multiple human ovarian cancer cell lines after 48 hours shows minimal cell death induction. **B**. PEITC [5 μM] induces significant cell death of multiple human ovarian cancer cell lines after a 24 hour treatment. Cell death percentage was determined using hemacytometry with Trypan blue exclusion to count both live and dead cells. * p < 0.01.

**Figure 3:**
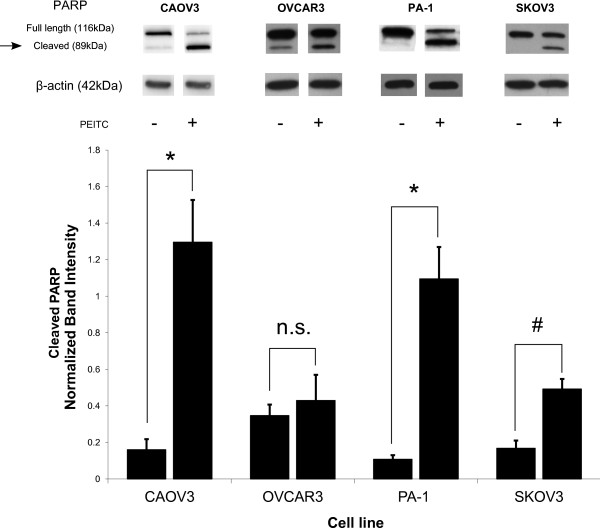
**Cause of cell death for PEITC treated cells.** PARP cleavage is increased by treatment with PEITC in multiple ovarian cancer cell lines, indicating death by apoptosis. Western blot for full length and cleaved PARP after 48 hours of treatment is displayed. Blots are representative images of band intensity. Bar graph represents cleaved PARP band intensity normalized to β-actin. * p < 0.01, # p < 0.05, n.s. = not significant.

When the same cell lines were treated with PEITC, their growth was also significantly inhibited (Figure 1B). While metformin is highly effective at reducing cell proliferation, cell death is not induced. On the contrary, PEITC was cytotoxic to many ovarian cancer cell lines (Figure 2). Cell death induced by PEITC was dose dependent and significantly higher than controls in the low micromolar range. Examination of PARP showed a shift from intact full length PARP to the cleaved form when cells were treated with PEITC (Figure 3). This shift suggests apoptosis as the primary cause of cell death for PEITC. PARP cleavage was not observed in cells treated with metformin over the same time period. This is consistent with the results showing a lack of metformin-induced cell death in the cell lines examined.

### ROS is increased with both metformin and PEITC

**Figure 4:**
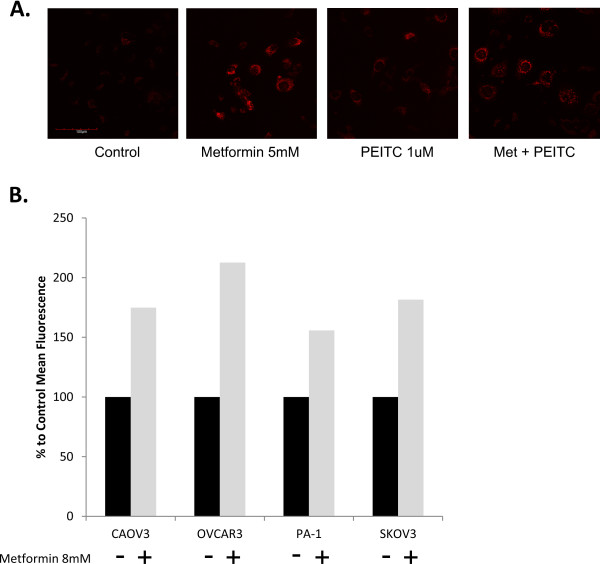
**Mitochondrial reactive oxygen species (ROS) increased with metformin and PEITC treatment.** A. Live cell confocal image of SKOV3 cells treated for 24 hours. MitoSOX, a mitochondrial specific superoxide detector, exhibits increased fluorescence in metformin and PEITC treated cells comparison to control treated cells. B. Mean fluorescence intensity from dihydroethidium (DHE) used to detect total cellular ROS. Cell lines were treated for 24 hours with or without metformin [8 mM] and DHE intensity was measured by flow cytometry.

Because previous research has found metformin to inhibit complex I and PEITC to inhibit complex III of the mitochondrial electron transport chain, ROS levels were examined as a potential mechanism for each drug's anti-cancer effects. Inhibition of complex I increases the aberrant flow of electrons to oxygen and creates superoxide within the mitochondrial matrix. Inhibition of complex III also increases superoxide production, but can generate these ROS species in either the cytosol or mitochondria because of its position in the mitochondrial membrane [18]. When live cells were treated with metformin or PEITC, they showed increased ROS as measured by MitoSOX (Figure 4A). MitoSOX is an ethidine based mitochondrial targeted probe that preferentially reacts with superoxide to produce a fluorescent product. Dihydroethidine (DHE) is a non-targeted ROS detector and can assess total cellular oxidative stress. In cells treated with metformin, fluorescence of DHE as detected by flow cytometery was also increased (Figure 4B). These results suggest that previously identified mechanisms of ETC complex inhibition by both metformin and PEITC results in an increase of ROS within the cancer cell.

**Figure 5:**
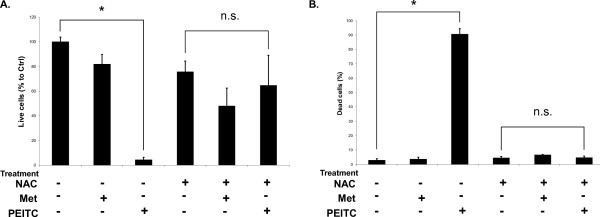
**NAC supplementation reverses PEITC cell death.A**. The effect of NAC supplementation on control (DMSO vehicle), metformin [5 mM], and PEITC [5 μM] growth inhibitory effects on PA-1 cell line after 24 hours. Metformin is relatively unaffected by the addition of NAC, while PEITC's growth inhibition is significantly reduced. **B**. The dead cell percent of PA-1 cells treated with control (DMSO vehicle), metformin, or PEITC treatment with and without NAC pre treatment. Metformin alone does not induce high amounts of cell death with or without NAC. PEITC's cell death is strongly reduced to levels similar to control when pretreated with NAC to act as an ROS scavenger. * p < 0.01; n.s. = not significant.

In order to determine the role of ROS in each drug's effects on cell proliferation or apoptosis, ROS scavenging compounds were used. When the ROS scavenger N-acetyl-L-cysteine was used to pretreat cancer cells before addition PEITC, cell death induction by the drug was completely reversed (Figure 5). This finding suggests PEITC is heavily dependent on ROS production and oxidative stress to induce apoptosis.

**Figure 6:**
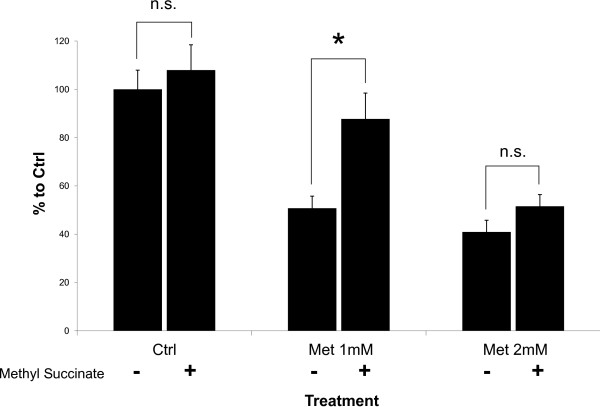
**Methyl succinate partially reverses metformin's effects.** When 12.5 mM methyl succinate is added to complete growth media, the 48 hour growth inhibition effects of metformin on PA-1 are reduced. Methyl succinate serves as an alternate energy source to glucose that bypasses complex I in the electron transport chain. Metformin at higher doses can overcome the reversal of growth inhibition provided by methyl succinate. * p < 0.01.

While PEITC-induced cell death is reversed by addition of a ROS scavenger, the effects of metformin on cell proliferation are not strongly affected. This may be due to metformin's general action of growth inhibition versus PEITC's induction of apoptosis. Metformin's complex mechanism, working through several pathways may also play a role. In situations where complex I inhibition does not produce high amounts of ROS to damage a cancer cell's mitochondria, metformin's action through AMPK and mTOR may still allow growth inhibition to be achieved. However, treatment of cells with methyl succinate partially reversed the ability of metformin to inhibit growth of PA-1 cells (Figure 6). Methyl succinate can bypass electron transport through complex I because it donates electrons directly to complex II of the mitochondrial electron transport chain. These results suggest inhibition of electron flow may be responsible for some of metformin's growth inhibition effects. As metformin concentration increases, the effect of methyl succinate is overcome, which is likely a result of metformin-induced activities such as AMPK activation.

### Metformin and PEITC combined are effective in a cisplatin resistant cell line

**Figure 7:**
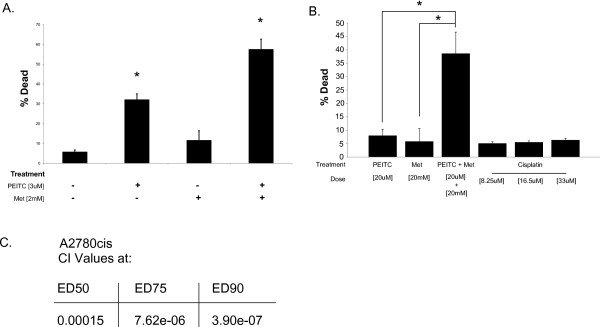
**Combination of metformin and PEITC.A**. Combination of low doses of metformin and PEITC on CAOV3 show an improved effect on cell death after 72 hours compared to either drug individually. Combination can reduce doses necessary to achieve high levels of cell death. **B**. In the cisplatin resistant ovarian cancer line A2780cis, metformin and PEITC work synergistically and are more effective than cisplatin. Cell death is significantly increased when metformin and PEITC are given together. Cisplatin, even at higher doses, is unable to produce similar cell death. **C**. Combination index (CI) values for A2780cis calculated using CalcuSyn software. CI values below 1 are considered synergistic combinations, with greater synergy as the value approaches zero. Metformin and PEITC are synergistic at equal ratio effective doses (ED). * p < 0.01 when compared to control treatment.

The results above indicate that metformin and PEITC function through different mechanisms and have different consequences on the growth and survival of ovarian cancer cells. Therefore we hypothesized that the two drugs would act synergistically when used in combination. In the cell line CAOV3, combination therapy significantly increased cell death over either drug individually (Figure 7A). Attaining the high levels of cell death observed for the combined drugs requires much higher doses with the individual drugs. Furthermore, this combination continues to be effective in the cisplatin resistant cell line A2780cis. Even high doses of cisplatin were not able to match the cell death obtained through metformin and PEITC combination in the A2780cis cell line (Figure 7B). A Combination index (CI) is a value used to quantify the degree of synergy or antagonism in drug combinations[19]. As a general guideline, CI < 0.7 is synergistic, 0.7 > CI <1.2 is additive, and CI > 1.2 is antagonistic. When determined using CalcuSyn software (BioSoft), the metformin and PEITC combination was found to be synergistic at several effective doses in the cisplatin resistant line A2780cis (Figure 7C). Calculated synergy is higher the closer the CI is to 0. With a CI of 0.00015 at ED50 metformin and PEITC doses, the combination is highly synergistic and increases with higher ED combinations. In this cell line, cisplatin remains ineffective at inducing cell death at doses as high as 33 μM or 10 μg/mL.

## Discussion

Expanding ovarian cancer therapies to include compounds such as metformin and PEITC is attractive for multiple reasons. Metformin has a broad safety profile, and while the mechanism is not exactly known, a tremendous amount of patient dosage data exists. The risk for lactic acidosis in metformin is minimal, with cases only commonly occurring with coexisting kidney dysfunction [20]. Similarly, PEITC's dosage safety can be partially inferred from cruciferous vegetable intake. A study of a defined watercress diet showed significant uptake of PEITC in humans [21]. A current phase I trial of PEITC in leukemia patients will further clarify safety and dosing.

Metformin's complex mechanisms of action may provide many difficulties to cancer cells attempting to counter its actions. Because metformin likely acts through multiple pathways, a cancer cell may be able to respond to the stress of one change, but still be affected by other alterations. Metformin's resistance to a variety of ROS scavengers, propyl gallate, butylated hydroxyanisole, Mito-TEMPO (data not shown) shows that metformin is not reliant on ROS production to inhibit cancer cell growth despite its actions on complex I. Still, complex I inhibition partially contributes to the cell inhibition as shown through the methyl succinate supplementation experiment. The methyl succinate prevention of metformin’s effect was diminished as metformin dose increased, suggesting metformin acts through multiple pathways as it accumulates within the cell. Higher doses of metformin may act through AMPK pathway activation through a still unknown exact mechanism. Likely, metformin's cellular effects are a combination of AMPK pathway activation with subsequent mTOR inhibition[22] and complex I partial inhibition increasing ROS.

Relevant dosing for metformin *in vitro* remains unclear. Most groups work in the low millimolar range, from 1–20 mM [3,22,23]. While this seems initially unattainable *in vivo*, metformin is not metabolized and has been found to be highly concentrated with the cells, especially within mitochondria [7]. Moreover, these concentrations are necessary for *in vitro* work to mimic the effects observed with *in vivo* studies where approved diabetic metformin doses have been used. Thus, in order to achieve the concentrated cellular metformin levels and study the potential changes that occur *in vivo* over longer periods of treatment, high doses over short time courses must be used *in vitro*.

While PEITC does not have the long historical use in humans that metformin has, its general safety can be inferred from diets high in cruciferous vegetables. Also, similar to metformin, PEITC has been associated with cancer prevention in several cohort studies [15,24].

Although PEITC is more effective at inducing cancer apoptosis, it is also more simply reversed by increasing ROS scavengers. Metformin's enhancement of PEITC could be due in part to depletion of reducing agents[25] needed to handle the ROS stress induced by PEITC. Additionally, metformin may synergize with PEITC through inhibition of complex I to provide extra mitochondrial oxidative stress, shifting the ROS balance towards cell death.

Additional therapies that utilize different mechanisms than cisplatin are especially attractive for ovarian cancer. The combination therapy of metformin and PEITC is interesting because of its effectiveness despite cisplatin resistance. Additional work is warranted to determine if this synergy is maintained with *in vivo* models. Current data suggests metformin and PEITC as a combination treatment have potential as a novel ovarian cancer therapy.

## Conclusions

Complex I inhibition is partially involved in metformin's growth inhibition of ovarian cancer, possibly by increasing ROS and sensitizing cancer to additional oxidative stress. PEITC induces ovarian cancer cell death by increasing ROS. When given together, metformin and PEITC show a synergistic increase in cell death in several ovarian cancer cell lines, including cisplatin resistant A2780cis cell line. This novel combination may be useful in combating the high mortality of cisplatin resistant ovarian cancers.

## Abbreviations

PEITC, Phenethyl isothiocyanate; ROS, Reactive oxygen species; ETC, Electron transport chain; NAC, N-acetyl-cysteine; DHE, Dihydroethidine.

## Competing interests

The authors declare they have no competing interests.

## Authors' contributions

DKC participated in the design of the study, performed the experiments, and drafted the manuscript. WKM participated in design and coordination of the study and helped to draft the manuscript. All authors read and approved the final manuscript.
